# Comparing Human Performance on Target Localization in Near Infrared and Long Wave Infrared for Cluttered Environments

**DOI:** 10.3390/s24206662

**Published:** 2024-10-16

**Authors:** Li Zhang, Mark Martino, Orges Furxhi, Eddie L. Jacobs, Ronald G. Driggers, C. Kyle Renshaw

**Affiliations:** 1 CREOL, College of Optics and Photonics, University of Central Florida, Orlando, FL 32816, USA; 2 Department of Electrical Engineering, University of Memphis, Memphis, TN 38152, USA; 3 Wyant College of Optical Sciences, University of Arizona, Tucson, AZ 85721, USA; 4 Department of Electrical and Computer Engineering, University of Central Florida, Orlando, FL 32816, USA

**Keywords:** infrared imaging, human perception, machine vision

## Abstract

In the context of rapid advancements in AI, the accuracies and speeds among various AI models and methods are often compared. However, a basic question is rarely asked: is AI better than humans, and if so, under what conditions? This paper investigates human ability to detect distant landmark targets under cluttered surroundings such as buildings, trees, and clouds in NIR and LWIR images, aiming to facilitate AI object detection performance analysis. Our investigation employs perception tests and a human performance model to analyze object detection capabilities. The results reveal distinctive differences in NIR and LWIR detectability, showing that although LWIR performs less effectively at range, it offers superior robustness across various environmental conditions. Our findings suggest that AI could be particularly advantageous for object detection in LWIR as it outperform humans in terms of detection accuracy at a long range.

## 1. Introduction

The rapid advancement of Artificial Intelligence (AI) profoundly impacts numerous domains, notably navigation, where it facilitates object recognition and localization. A recent study has demonstrated AI’s capability to recognize distant objects and geo-localize them [1,2,3,4,5], making autonomous driving possible. Such AI should also work at night, when Long Wave Infrared (LWIR), with wavelengths ranging from 8 to 14 μm, is extensively used. In this case, having a human’s target detection model to compare with AI’s performance is crucial, especially for a long target to sensor distance, harsh environments, and fast-moving objects. LWIR imaging, as one type of thermal imaging, encounters challenges like low resolution and a low signal-to-noise ratio due to microbolometer sensor arrays. They reduce image quality and potentially degrade human performance. Conversely, these limitations could offer Artificial Intelligence (AI) systems an advantage over humans as rapidly evolving AI might be better equipped to interpret and analyze lower-quality images [2,3].

In light of these considerations, our paper aims to study human performance in target detection within cluttered, real-world scenarios to provide a comparison to the AI results. We fit the perception test results to a human performance metric, targeting task performance (TTP) to determine and validate some empirical constants. We also include Near Infrared (NIR) images in the test as a comparison. By employing perception tests that span both NIR and LWIR spectrums, we aspire to provide a comparative analysis that not only highlights the strengths and weaknesses of each band but also outlines potential improvements for integrating AI into vision-based navigational systems. Due to a lack of AI range performance data from existing publications, we use our previous AI object detector training results to compare with the human performance model from this work. Note that our AI object detector was trained with a limited dataset size and network complexity, and other well-established detectors may perform better in terms of range, although they are rarely evaluated in this way.

## 2. Theory

A model that quantifies imaging system performance for the task is essential to compare the target detection ability of humans and AI. The TTP metric, developed by the U.S. Army, is a robust model that predicts human performance for military search tasks where the target is resolved. The TTP is based on target–background contrast $c_{tgt}$ and the imaging system’s contrast threshold function $CTF_{sys}$. Every part of the system is modeled to compute these quantities, including the illumination, target characteristics (reflectivity, emissivity, and size), atmosphere (attenuation, scintillation), optics, focal plane, display, and the human eye [6,7,8]. The TTP is given by the following:

(1) $$TTP=\int_{\xi_{low}}^{\xi_{cut}}{\left[\frac{C_{tgt}}{CTF_{sys}\left(\xi \right)}\right]}^{1/2}d\xi ,$$

where ξ is the spatial frequency and the integration is over all frequencies where $CTF_{sys}\left(\xi \right)<c_{tgt}$. The target contrast is given by the following:

(2) $$c_{tgt}=\frac{\sqrt{{\left(\mu_{tgt}-\mu_{bg}\right)}^{2}+\sigma_{tgt}^{2}}}{\mu_{tgt}+\mu_{bg}},$$

where $\mu_{tgt}(\mu_{bg})$ is the target (background) mean signal and $\sigma_{tgt}$ is the variance of the target signal. In simplest form, the $CTF_{sys}$ can be written as follows:

(3) $$CT{F_{sys}}^{2}\left(\xi \right)=\frac{CTF^{2}\left(\xi \right)}{MTF^{2}\left(\xi \right)},$$

where MTF(ξ) is the modulation transfer function of the imaging system that affects the image displayed to the human observer, including blur from the optics, detector sampling pitch and size, and display resolution. However, it does not consider aberrations from the optics and assumes diffraction-limited spot size. The CTF(ξ) is the contrast threshold function of the human eye which, in principle, depends on the specific observer; in practice, we use a representative CTF(ξ) for an “average” observer which has been developed through extensive perception studies. We use the standard CTF(ξ) described in Ref. [6].

When an image containing the target is taken with the given system, the total number of resolved cycles (V) is given by the following:

(4) $$V=\frac{\sqrt{A_{tgt}}}{R}TTP,$$

where $A_{tgt}$ is the cross-sectional area of the target viewed by the imager, called the characteristic dimension, R is the range to target, and $\frac{\sqrt{A_{tgt}}}{R}$ approximates the angular subtense of the target as a geometric mean. The probability that a human can detect the target ($P_{D}$) is related to V according to the following:

(5) $$P_{D}=\frac{{\left(\frac{V}{V_{50}}\right)}^{A+B\left(\frac{V}{V_{50}}\right)}}{1+{\left(\frac{V}{V_{50}}\right)}^{A+B\left(\frac{V}{V_{50}}\right)}},$$

where the three constants ($V_{50},\;A$, and B) are empirically derived for a given scenario, such as recognizing drones in the sky, identifying trucks on the ground, or, in this case, detecting towers on the horizon. The quantity $V_{50}$ reflects the task difficulty as it describes the number of resolved cycles needed to achieve 50% $P_{D}$. The $V_{50}$ value changes for different tasks such as detection, recognition, and identification (each has a higher task more difficult than the previous). For the purpose of this paper, we focus on the detection task, which is the simplest. This means that we are not discriminating against different types of towers. Sometimes objects like light or power posts might cause confusion or false alarms, but they are not counted for our purpose. The coefficients A and B describe the speed at which the logistic function decays. A set of typically used empirical values are A=1.51 and B=0.24 [7].

## 3. Methods

### 3.1. Hardware Setup

A multi-spectral camera payload was integrated onto a 12″ Zaber motorized gimbal on top of a Jeep Gladiator. The payload and gimbal were housed inside a weatherproof enclosure with a removable lid to allow for imaging around the vehicle using all of the spectral bands available. Details of the general setup can be found in Ref. [8]. Only the LWIR and NIR cameras are used in this study. The LWIR camera is a FLIR BOSON microbolometer used with an 18 mm fixed focal length lens. It has 640 × 512 pixels (i.e., VGA format) on a 12 µm pixel pitch, giving a field-of-view (FOV) of approximately 25°. The NIR camera is an Alvium 1800 series camera used with a 24 mm lens; a 700 nm long pass filter was installed inside the camera to cut off the short wavelength response of the silicon sensor. The NIR camera has 4128 × 3008 pixels on a 3.45 µm pitch, giving an FOV of approximately 27°. These cameras are approximately FOV-matched, but the resolution of the NIR camera is much larger. For the perception study presented here, we down sampled the NIR imagery to match the VGA format of the LWIR camera. Table 1 below shows the spec. of the camera and lens used.

The Jeep is also equipped with a VectorNav 300 dual-GPS inertial navigation system (INS), which provides GPS location and vehicle orientation with a high degree of accuracy. A Neousys NUVO-8208GC rugged edge computer was installed in the vehicle and used to control the hardware and collect data. Custom MATLAB and python applications were developed to control instruments, track towers, and collect data from an operator station in the passenger seat.

### 3.2. Data Collection

The vehicle mounted system was used to take images of various types of towers with different shapes and sizes over a large span of distances. The tower data were precompiled from digital obstacle files (DOFs) for the region that are maintained by the federal aviation administration (FAA); this appears to include all towers > 300′ in height and many shorter towers, but not all. To help the operator collect data on the desired towers, the control software loads these data and plots them onto a street map along with the vehicle’s current location and orientation. A separate panel in the software shows imagery from a desired camera and provides controls for steering the gimbal while recording data from all cameras. A tracking interface was implemented part way through the data collection to allow the operator to select a designated target (i.e., tower) and continuously track it.

Imagery collected without target tracking was manually correlated to the FAA tower data during post-processing using metadata recorded alongside the imagery. The metadata includes GPS time, frame number, vehicle location, vehicle velocity, vehicle orientation, and gimbal orientation. When tracking is used, the metadata also contains the FAAs unique tower ID for the tower being tracked.

All imagery was then manually examined to determine whether there was one or more tower(s) in the image. Around each tower, a rectangular bounding box is drawn to record its location, width, and height. This ground truth and the metadata are used to correlate towers in the imagery with towers in the database; after correlation, we obtain the tower location from the database and calculate distance R from the known vehicle location. Some outlier cases, where the target is at the very edge of the FOV and where the bounding box cannot be well defined without including too many other objects, such as power post streetlamps and trees, were removed.

### 3.3. Perception Test

A perception test was designed so that the $V_{50}$ of this task can be determined by fitting the data for LWW and NIR separately. An app was written in MATLAB to display the images and record participant responses. The tabletop display used for the test has a maximum brightness of 250 cd/m² and a pixel pitch of 0.155 mm. The format and FOV of both image sets were matched as described above. NIR images were rescaled by a factor of 0.165, so their sizes are comparable with LWIR images. In total, 11 participants took the perception test, where they were asked to locate all the towers that they see in a total of 150 images by clicking on them. Each image contains 0 to 4 tower(s), and the same tower may appear in many images taken at different locations. The images are displayed in random order and flipped horizontally every other image to avoid repetition. Participants were allowed to modify their response for one image before moving to the next. They were not timed and were seated at 50 cm from the display. Responses were compared to tower bounding boxes to determine detection probabilities. For each participant, a 0 or 1 score is assigned to each tower bounding box. For the scores, 0 is for miss and 1 is for correct detection. False alarms are not counted. The total number of towers in all images is 145, each with a corresponding rate of detection calculated by averaging the scores across 11 participants.

### 3.4. NVIPM Analysis

**Figure 1:**
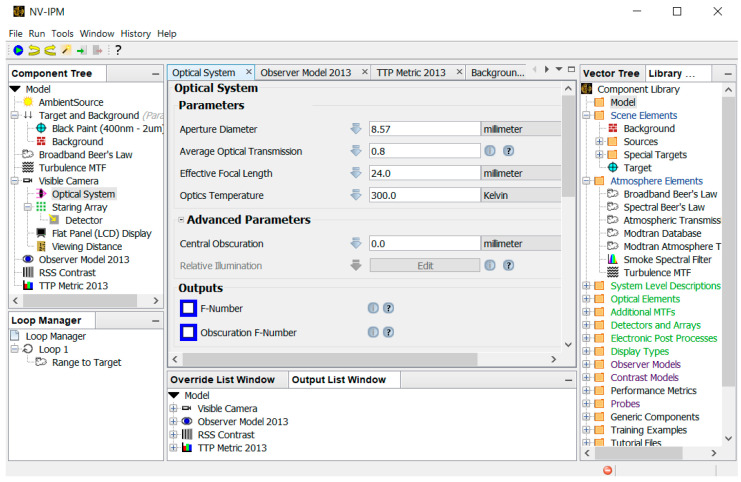
Snapshot of NV-IPM software user interface showing the sub-model of the optical system for the NIR case.

In this study, we fit the perception test results to the TTP metric model with the help of the Night Vision Integrated Performance Model (NV-IPM) [9], a software developed by the U.S. Army’s Night Vision and Electronic Systems Directorate (NVESD). It helps calculate metrics such as TTP and estimate human probability of detection for a given system, environment, observer model, and task difficulty. We employed a combination of pre-existing NV-IPM models to obtain TTP values for NIR and LWIR as a function of range. The models include the target and background model, broadband Beer’s law for atmospheric transmission and scattering, imaging optic, sensor, constant gain level, Root Sum of Squares (RSSs) contrast, display, observer, and TTP metric model. Figure 1 shows a snapshot of the NV-IPM software version 1.10 when modeling for the NIR imaging system.

Note that the broadband Beer’s Law requires little to no scattering and homogeneity in the atmosphere, which is sometimes not the case for the hot and humid environment in Florida, especially on sunny days. Assuming that the atmosphere was mostly homogeneous when we took the data, Beer’s Law works well for LWIR, where the scattering effect is minimal, but not as well for NIR, where scattering is dominant. This might lead to mismatch between model output and observation for the contrast calculation in the results section.

For NIR, we input the actual quantum efficiency of our sensor to NV-IPM from 0.7 to 1 μm. The reflectivity of the towers in NIR is generally unknown and varies depending on the paint or material used. It is set in NV-IPM as 0.14 to better fit our contrast data. Most of the time, the tower is under cloud background due to cloudy weather during data collection dates. Thus, we chose cloud as the background when modeling, which, according to measurements taken by S. Twomey and T. Cocks [10], has an average reflectance of approximately 0.8 at 0.75 μm.

For LWIR, our camera was not calibrated to measure target and background temperatures. As a result, we made estimates about background (i.e., sky) temperature based on local weather during the day of data collection and then used the actual contrast in the image to back calculate target temperature and atmospheric transmission. Details are discussed in our previous work Ref. [11]. A linear contrast stretch was performed before presenting LWIR imagery to the test participants; the lowest 5% and highest 95% of the pixel values were mapped to the 8-bit display range. A constant gain level model was used in NV-IPM to account for it. The amount of gain was set to be 0.21, so the simulated result fits the contrast of the actual stretched images.

## 4. Results

**Figure 2:**
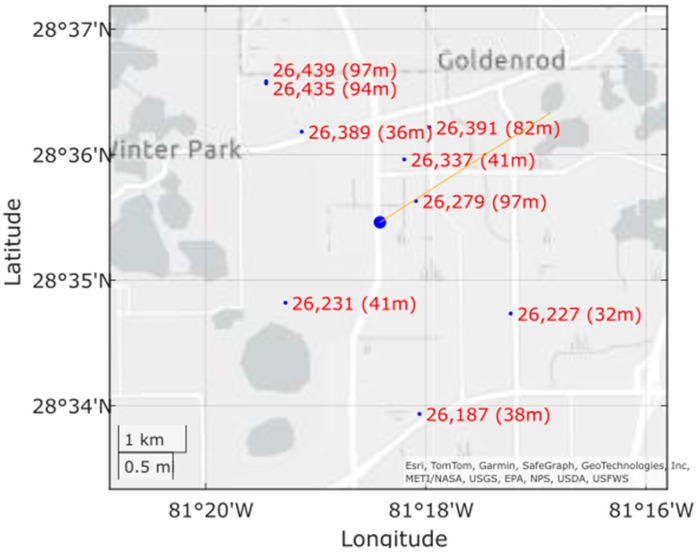
Map of the region in Orlando around the data collection vehicle containing towers (small blue dots with red ID number and height), vehicle location (big blue dot), and gimbal orientation (orange line extending from the big blue dot).

Figure 2 shows the real-time map displayed in the user interface to aid data collection. It is also used offline to verify tower ID in imagery. The location of the data collection vehicle is indicated by a large blue dot on the map, and the orientation of the gimbal is shown by an orange line extending from the vehicle’s location. Tower locations are also shown as small blue dots alongside their unique FAA ID number and their height. This is updated in real time as the vehicle moves.

**Figure 3:**
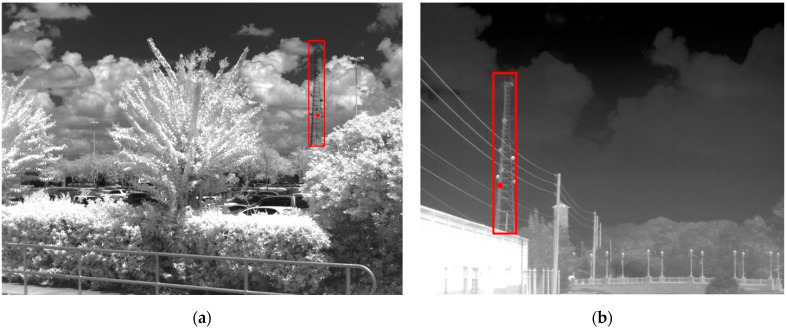
Image in NIR (**a**) and LWIR (**b**) of the same tower taken at different locations and target ranges. In NIR, the tower is dark under a bright background, whereas in LWIR, the tower is bright under a dark background. Each participant’s response is represented by a red dot. In the above cases, both dots lie within the rectangular ground truth bounding box and are counted as a correct detection.

Example NIR and LWIR images provided in the perception test are shown in Figure 3. Note that contrast stretch is performed on raw LWIR images to produce the ones displayed here and in the perception test. A bounding box has been added around the target, but this is not present during the test. The participant must click within the bounding box for a correct detection to be made. A red dot is shown, representing where one participant clicked so the participants can see and modify their responses.

**Figure 4:**
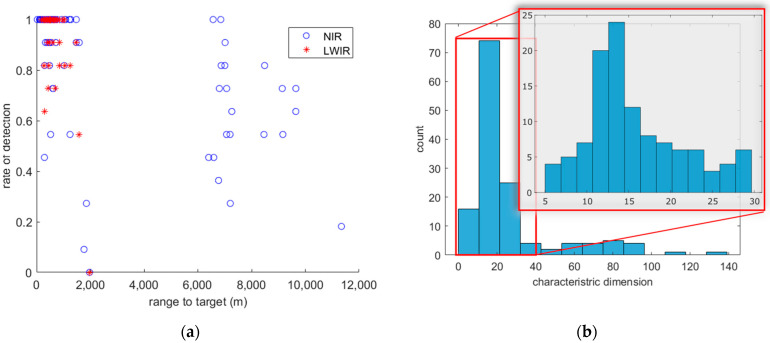
(**a**) Raw data of detection results for NIR and LWIR(averaged for all the responses for the same tower case) plotted versus range. (**b**) Histogram of tower character dimensions $\sqrt{A_{tgt}}$.

Identifying towers in images taken from the street level is a complex task by nature. While driving the data collection vehicle, we can only obtain a clear view of a few towers at certain locations; otherwise, the towers are obstructed by buildings or vegetation. Additionally, each tower that we have access to has a unique shape and height, leading to different characteristic dimensions. This complexity obscures the relationship between detection probability $P_{D}$ and range to target R, as shown in Figure 4a. Limited by tower height, road status, and buildings, we were unable to obtain towers in urban settings from a distance further than 2 km. Some radio towers in the suburbs are exceptionally tall (400~600 m) and can be seen from more than 6 km away. However, they were surrounded by tall trees, and few public roads lead to or get close to them. These real-world limitations resulted in a gap in the data within a range of 2–6 km.

Figure 4b shows the distribution of characteristic dimension $\sqrt{A_{tgt}}$. The target area is approximated by the area bounded by the rectangular ground truth box. Despite some extreme cases, most characteristic dimensions computed this way are between 5 and 30 m, with a median of 15.86 m.

This distribution indicates that the actual target area visible in the imagery needs to be considered when modeling for $P_{D}$. The towers with large characteristic dimensions are the tall radio towers in the suburbs that are more than 6 km away and are only visible in NIR images. By calculating apparent target characteristic dimension in each specific image (square root area of the target that is visible in the image, not counting obstructions), we can sort the seemingly random raw data into processed data that show a clear trend.

**Figure 5:**
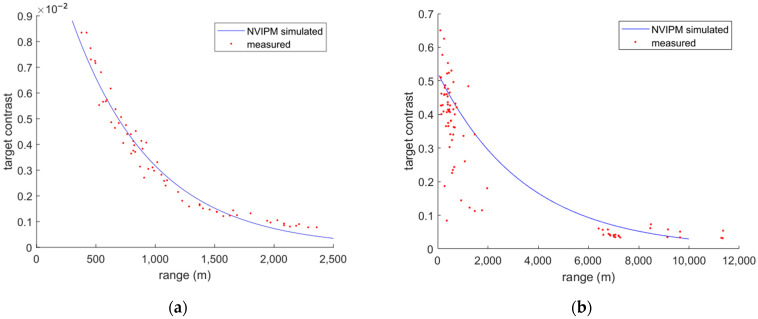
Contrast of towers in LWIR (**a**) and NIR (**b**) under cloud background plotted against observation range. Measured contrast (red points) is calculated for each tower in each image. LWIR results are taken from previous work [11], and NIR results use new data. Simulated curves are obtained from NV-IPM and fitted to the measured data using target temperature/reflectance and atmospheric transmission as fit parameters.

Figure 5 plots the contrast in the images between the target and background for LWIR and NIR compared with NV-IPM simulations (with settings detailed in Section 3.4). A region of interest (ROI) is selected around a clear (i.e., uncluttered) portion of each tower under cloud background; the box is sized to include at least twice as many background pixels as tower pixels. Then, tower contrast is calculated for the raw images using Equation (2), the same equation that the NV-IPM uses.

For LWIR, Equation (2) is simplified for negligible $\sigma_{tgt}$ because there are few variations in the raw image and the target temperature is expected be uniform. For the same reason, we use the maximum value in each horizontal line in the ROI and average overall lines to obtain $\mu_{tgt}$. For NIR, more details of the tower are visible and $\sigma_{tgt}$ is calculated. Pixel values within 0.7 standard deviation of the minimum value in each horizontal line are counted as a tower. Their mean and standard deviation are used as $\mu_{tgt}$ and $\sigma_{tgt}$. The background pixels are selected after masking off the tower. This normalization scheme does not account for background variation or noise but is what NV-IPM uses. We adopt this so that we can fit our data to NV-IPM’s model.

For LWIR, NVIPM simulation used a background temperature of 298 K, target modeled as a black body with a temperature of 306.2 K, and atmospheric transmission of 0.23 $km^{-1}$, which is a value fitted to our contrast data obtained from raw LWIR images. The actual tower temperature under solar loading and its thermal emissivity are not known; for convenience, we fix emissivity = 1 (i.e., black body) and model temperature to control the thermal signature. For NIR, the contrast is simulated using the built-in solar illumination with target (background) reflectivity of 0.25 (0.8) and atmospheric transmission of 0.75 $km^{-1}$; note that the high background reflectivity is due to clouds that were always present behind the selected tower regions. Larger variability is observed in NIR data, presumably due to variations in paints that give different reflectivities, atmospheric scattering, variations in illumination, and types of cloud backgrounds; these same clouds were not visible (i.e., cloud looked uniform and blended with clear sky) in the LWIR imagery. In both cases, Beer’s law was used to model atmospheric attenuation, which does not account for the potentially high scattering in NIR, which might contribute to the mismatch between model and data. The temperature, reflectivity, and atmospheric transmission values were selected manually to provide a good fit to the observed contrast so that we can match this scenario in an NV-IPM model and compare real detection results to the model predictions.

**Figure 6:**
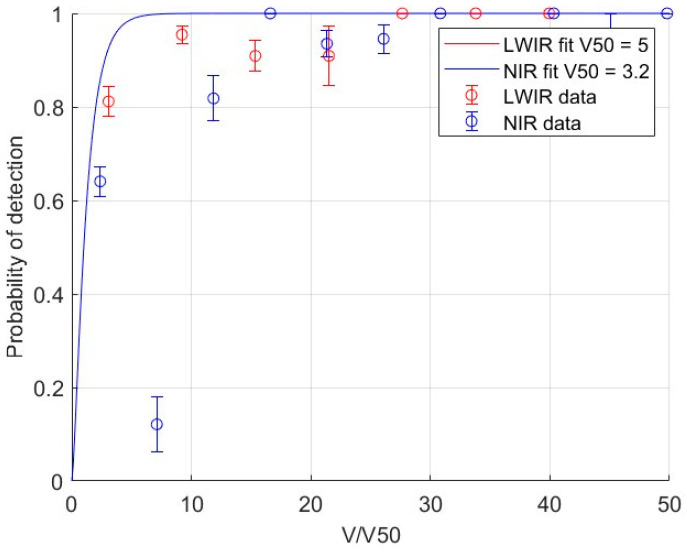
Rate of detection from the perception test plotted as a function of $V/V_{50}$. $V_{50}$ is a parameter used to fit the data to the detection probability ($P_{D})$ model presented in Section 2 plotted as solid lines. Data and fits are shown for both NIR and LWIR results. $R^{2}$ of the fits are 0.94 for LWIR and 0.91 for NIR.

Figure 6 summarizes the empirical detection probability data from the perception test but reported as a function of $V/V_{50}$, where $V_{50}$ is fit using the model described in Section 2. Here, the raw data were binned to yield comparable sample numbers in each bin; the average of each bin is shown as a data point, while the standard deviation of each bin is shown as an error bar. The results are shown for both LWIR and NIR data. The $P_{D}$ from the model (i.e., Equation (5)) is plotted for the resulting $V_{50}$ in each band and shows good agreement with the perception data; note that these overlap when plotted in units of $V/V_{50}$. The default A and B parameters built into NV-IPM yield satisfactory results. Including these as fit parameters did not provide substantially better match to the data, so we elect to fix these to the default values, which work well for the archetypal tank detection task.

## 5. Discussion

We must know (or estimate) the size of a given tower in order to relate its detection probability to the model described in Section 2. While the FAA database provides tower height, the width is generally unknown, and the bottom portions of towers are usually obscured. Instead, we measure the angular subtense of the unobscured portion of the target based on its size in the image. Specifically, the characteristic dimension is calculated for each tower in each image as follows:

(6) $$\sqrt{A_{tgt}}=R*\sqrt{\tan^{-1}\left(h*ifov_{v}\right)*\tan^{-1}\left(w*ifov_{h}\right)},$$

where $ifov_{v}$ and $ifov_{h}$ are the vertical and horizontal fields of view per pixel. These are equal due to the square pixel format and $ifov_{v}=$ $ifov_{h}=p/f$, where f is the lens focal length and p is the native (LWIR) or down sampled (NIR) pixel pitch. h and w are the target height and width in the ground truth data bounding only the visible part of the tower. Because the bounding box is rectangular, this is a still crude approximation that does not consider the tower shape (lattice-type towers will be bigger at the bottom, monopole towers will be smaller along entire length, etc.).

By fitting Equation (5) to our perception test data, we obtain $V_{50}$ for LWIR and NIR using the default coefficients A = 1.51 and B = 0.24. The resulting $V_{50}$ are 6.7 and 5.5 for LWIR and NIR, respectively. The data generally follow the model shape despite the intrinsically large variability in human perception studies with limited sample size. Both datasets, but particularly the NIR data, show consistently lower probabilities than the model at large V numbers. At large V, the model indicates that the task has become so easy that $P_{D}$ should be nearly 100%; in the real imagery, the presence of clutter and confusers (i.e., telephone poles, light posts, etc.) resulted in lower detection levels.

Recall that $V_{50}$ describes the number of resolved cycles required for observers to detect the object 50% of the time. Alternatively, this can be described in terms of $R_{50}$, where $R_{50}$ is the distance where detection probability falls to 50% and is given by the following:

(7) $$R_{50}=\frac{\sqrt{A_{tgt}}}{V_{50}}TTP.$$

This yields $R_{50}$ = 3.38 km (1.37 km) for NIR (LWIR) and corresponds to an increase in range performance by a factor of 2.5.

In Figure 6, the $P_{D}$ is plotted as a function of the range for a fixed tower characteristic dimension of 10 m, which is close to the median value observed in Figure 4b. The fitted $V_{50}$ are used for each band along with default exponent values (A & B). We see that even though the NIR image is rescaled to almost the same resolution as the LWIR image, it still has much better range performance. LWIR $P_{D}$ plunges, starting from approximately 1 km, while the NIR $P_{D}$ decreases slowly, even after LWIR $P_{D}$ reaches 0. This is consistent with the increased atmospheric transmission for the NIR that we discussed in Section 4 based on the empirical contrast variation observed in the dataset (c.f. Figure 5). Contrary to our expectations that longer wavelengths typically propagate through the atmosphere better, we see very low contrast in our raw LWIR image. This resulted in a fitted transmission of 0.23 $km^{-1}$ using broadband Beer’s law, which is much smaller than the suggested value for good weather (0.75~0.95) per NV-IPM help documentation . Due to the effects from defects or dust on the optics, the unexpected low responsivity of the microbolometer array could contribute to this, but it is expected to be small. The major cause of the low contrast we see in LWIR images should be low atmospheric transmission due to water vapor absorption in hot and humid environments. This is supported by Refs. [12,13], where significantly increasing water vapor absorption and decreasing range performances for LWIR are seen as humidity and temperature increase. The value 0.23 $km^{-1}$ is also close to the MODTRAN simulation result of 0.31 for 100% humidity environments [14]. NIR, however, is not influenced by humidity and temperature that much. Of course, NIR tower detection will only work during the daytime, unless one relies on visual beacons or strobes that are often placed on towers to prevent aircraft collisions. In contrast, LWIR tower detection can operate day or night. For NIR, the transmission that best fits our data is 0.75 $km^{-1}$, which is a typical value for good weather scenarios.

**Figure 7:**
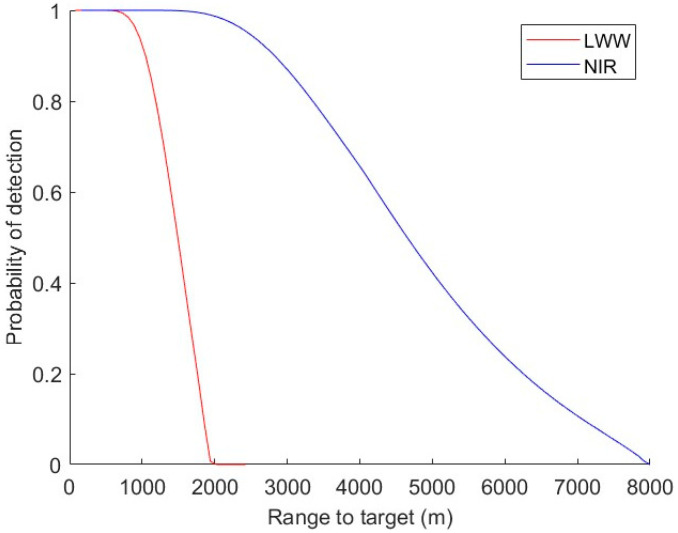
PD as a function of range for a tower of $\sqrt{A_{tgt}}=14$ in LWIR and in NIR.

**Figure 8:**
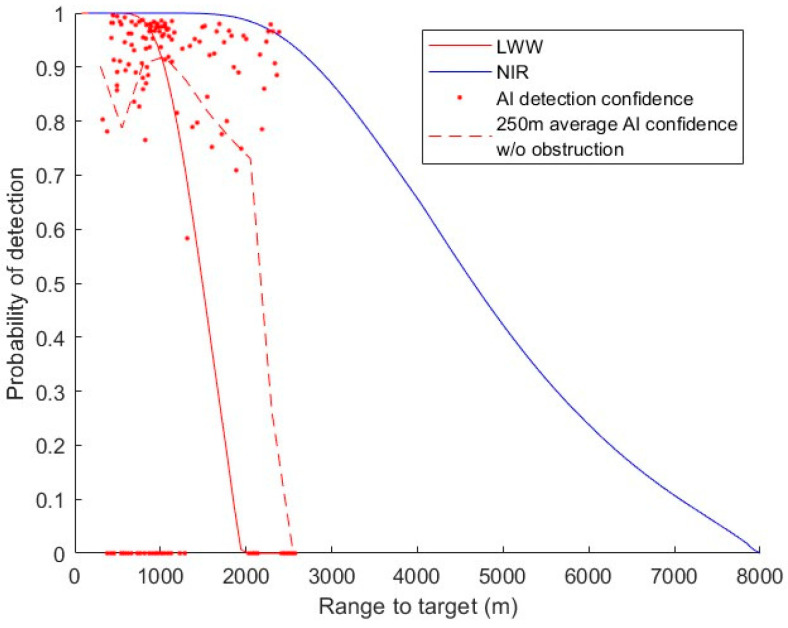
AI detection results for LWIR images plotted with human PD from Figure 7. The red dashed line is the average AI detection confidence for the cases where the target is not obstructed by other objects like power cables, traffic lights, and trees. The AI results are taken by our previous study Ref. [11].

We previously trained an object detection CNN on LWIR images and obtained approximately 90% accuracy on tower targets that are 2.5 km away [11]. We used YOLO v4 and 264 LWIR images to train our model. The dataset was balanced to contain approximately 10% images, where the towers are significantly more difficult to spot than the others for both the training set and validation set. The validation mAP was 91%. The detector was tested first with 90 images, some of which contained no tower. From there, the model missed 5 towers and had 7 false alarms out of 90, totaling 87% accuracy (not considering detailed bounding box parameters). It is then tested with a few image sequences taken while the vehicle is driving on roads or highways, capturing a total of 157 towers. A total of 49 of the towers are missed by the detector, and most of them had trees or power cables in the background or foreground. From the results of the second test, a plot with confidence vs. range is generated and overlayed with Figure 7 to generate Figure 8. Confidence here is model output prediction confidence and is set to 0 for missed tower. False alarms are not included. Part of the results where towers were under clear sky background (no apparent trees or power cables in the background or foreground) are averaged every 250 m to produce the dashed line.

This AI tower detector trained for LWIR performs better than human observers at a range longer than approximately 1100 m for the cases where the targets are unobstructed or obscured. This suggests that AI can perform significantly better than human observers on thermal images, particularly when they are limited by contrast rather than resolution. Further training and investigation of NIR imagery can help facilitate this comparison. As a reference, another existing AI using convolutional neuro-network (CNN) achieved 93% accuracy on a dataset containing mid-wave infrared (MWIR) images with vehicles as targets, where the range to target was 3~5 km [3].

## 6. Conclusions

This study has contributed insights into the human capability of detecting targets in NIR and LWIR imagery under cluttered backgrounds from a long range. Through perception tests and the application of TTP metric and NV-IPM, we confirm that the empirical exponent parameters are still valid for our targets under cluttered environments. While NIR and LWIR imagery gives similar performances in short distances, they start to vary significantly after approximately 1 km, where NIR images provide a much better range performance compared to the LWIR images. Note that this result accounts for the scaling of the NIR image, so that its resolution is comparable to the LWIR image. As thermal imagery, LWIR is immune to changes in illumination and background conditions, but it lacks shadow and sharpness, which help with target detection. LWIR transmission also suffers from high humidity of the environments where our data were collected. As a result, NIR systems can provide much higher TTP (resolved cycles per milliradian) than LWIR systems under the same atmosphere conditions. However, the task of finding the target under cluttered background is also more difficult in NIR because a lot of objects in the background that are similar to the target in shape are clearly visible. These include streetlights, power posts, buildings, and trees. It is much harder to find the tower under a background filled by these tower-like shapes than under a background of detail-less shapes, which is the case for LWIR images. Due to this trade, the $V_{50}$ values, often referred to as task difficulty, of NIR and LWIR are close.

The findings of this paper set a cornerstone for future AI development and assessment. AI systems have high potential in enhancing target detection performance under conditions where human performance is degraded by sensor limitations or environmental factors. Future research should focus on developing the AI model, comparing with the results of this paper, and extending these findings by incorporating a broader range of environmental variables and more diverse target types. This could further enhance our understanding of the operational limits of human and AI systems in target detection tasks, leading to more robust and adaptable imaging and detection technologies.

## Figures and Tables

**Table 1:** Spec. of the lens and camera used for data collection.

Type	Lens EFL (mm)	F-Number	Pixel	Pitch (μm)	Spectral Band (μm)
LWIR	18	1.0	640 × 512	12	7.5~13.5
NIR	24	2.8	4128 × 3008	3.45	0.75~1.1

## Data Availability

The datasets presented in this article are not publicly available due to privacy and technical reasons but may be obtained from the authors upon reasonable request.
